# The alternatively spliced diacylglycerol kinase gamma-Δ exon13 transcript generated under hypoxia promotes glioblastoma progression

**DOI:** 10.32604/or.2024.055102

**Published:** 2025-04-18

**Authors:** MING YANG, LIANGZHAO CHU, SHUKAI LIN, HAN PENG, NIYA LONG, KAYA XU, HUA YANG, FENG HAN, JIAN LIU

**Affiliations:** 1 Department of Neurosurgery, The Affiliated Hospital of Guizhou Medical University, Guiyang, 550025, China; 2 Department of Neurosurgery, Sanya Central Hospital (Hainan Third People’s Hospital), Sanya, 572000, China

**Keywords:** Glioblastoma (GBM), Hypoxia, Diacylglycerol kinase gamma (DGKG), Alternative splicing

## Abstract

**Background:**

Glioblastoma (GBM) is one of the most malignant types of central nervous system tumors. Oxygen deprivation in the tumor microenvironment is thought to be an important factor in promoting GBM progression. However, the mechanisms of hypoxia-promoted tumor progression remain elusive.

**Methods:**

Alternative splicing of diacylglycerol kinase gamma (DGKG)-Δ exon13 was amplified and verified by PCR-Sanger sequencing. The functions of DGKG and DGKG-Δ exon13 were analyzed by Cell counting kit-8 (CCK-8), Transwell, Matrigel-transwell experiments, and *in vivo* orthotropic GBM animal models. Transcriptome analyses were done to find out the regulated genes.

**Results:**

In this study, we found that a new transcript DGKG-Δ exon13 was generated in GBM under hypoxia via alternative splicing. Moreover, the results of CCK-8, Transwell, and Matrigel-transwell experiments showed that the proliferation, migration, and invasion abilities of U87-MG and T98G were decreased after DGKG knockdown. Compared to wild-type DGKG, DGKG-Δ exon13 overexpression significantly promoted cellular proliferation, migration, and invasion abilities in GBM. Furthermore, *in vivo*, orthotropic GBM animal models analysis showed that the tumor volumes were much smaller in the DGKG knockdown group. However, the tumor sizes in the DGKG and DGKG-Δ exon13 rescue groups were restored, especially in the DGKG-Δ exon13 group. Transcriptome analysis revealed that MORC1, KLHDC7B, ATP1A2, INHBE, TMEM119, and FGD3 were altered significantly when DGKG was knocked down. IL-16, CCN2, and EFNB3 were specifically regulated by DGKG-Δ exon13.

**Conclusions:**

Our study found that hypoxia-induced alternative splicing transcript DGKG-Δ exon13 promotes GBM proliferation and infiltration, which might provide a new potential target for the clinical treatment and diagnosis of GBM.

## Introduction

Glioblastoma (GBM) is a malignant tumor of the central nervous system that originates from glial cells [1–5]. The presence of the special blood-brain barrier along with inter- and intra-tumor heterogeneity make GBM one of the most malignant types of tumors [6–8]. GBM is characterized by high recurrence rates and poor prognosis, with a five-year survival rate of only 5%, and the rates of disability and mortality due to GBM are high [9,10]. Therefore, understanding the pathogenesis of GBM is crucial for advancing its treatment and diagnosis.

Hypoxia is regarded as an important factor in the development of GBM [11,12]. The partial pressure of oxygen can drop to as low as 1% in severely hypoxic regions, and GBM tumors often exhibit rich microvasculature and necrosis, which are closely associated with hypoxia. Hypoxic conditions induce tumor cells to undergo alternative splicing, which generates new proteins that promote proliferation, migration, invasion, and angiogenesis, thereby allowing tumor cells to adapt to the tumor environment [13–15]. Therefore, understanding the mechanism by which hypoxia promotes the development of GBM is urgently necessary to develop more effective treatment strategies that can improve the quality of life and extend the duration of survival of patients with GBM [16–18].

The diacylglycerol kinase (DGK) protein family produces phosphatidic acid (PA) by phosphorylating diacylglycerol (DAG) [19,20]. DAG and PA are important second messengers and play important regulatory roles in signal transduction [21,22]. The DGK gamma (DGKG) enzyme belongs to the type I subfamily of DGKs, and previous studies have demonstrated that hypoxia-inducible factor 1-alpha (HIF-1α) can bind to the promoter regions of DGKG to activate its transcription under hypoxic conditions. The upregulation of DGKG promotes tumor progression by inducing the secretion of transforming growth factor-beta 1 [23,24]. The upregulation of DGKG has also been observed in acute myeloid leukemia, hemangioblastomas, and colorectal cancer [25–27]. Recent studies have demonstrated that the new transcript DGKG-Δ exon13 is significantly elevated in GBM [28]. However, the expression and molecular mechanism underlying the mode of action of DGKG and DGKG-Δ exon13 in GBM have not been reported. Therefore, the present study aimed to investigate the functions of DGKG in the development of GBM, which can aid in identifying potential novel targets for the treatment of GBM and improving prognosis.

## Materials and Methods

### Cell culture

U87-MG (Cat. HTB-14, RRID: CVCL_0022, American Type Culture Collection (ATCC), USA,), T98G (Cat. CRL-1690, ATCC, USA), A172 (ATCC, Cat CRL-7899, RRID: CVCL_0131) and U251-MG (Cat. TCHu58, RRID: CVCL_0021, National Collection of Authenticated Cell Cultures, China) human glioma cell lines were purchased from Fuheng Biotech Co., Ltd. (Shanghai, China) and maintained in minimum essential medium (MEM) (Cat. KGM4150N-0500; KeyGen, Nanjing, China) or Dulbecco’s modified Eagle’s medium (DMEM) (Cat. KGM12800N-500; KeyGen, China) supplemented with 10% fetal bovine serum (FBS) (Cat. A5256701; Gbico, Brazil) and 1% Penicillin-Streptomycin (Cat. SV30010; Hyclone, USA) at 37°C in an atmosphere of 5% CO_2_. The cell lines were tested free of Mycoplasma.

### Polymerase chain reaction (PCR) and sanger sequence

Analysis of the expression of the wild-type DGKG and alternatively spliced DGKG-Δ exon13 in U87-MG and T98G cells by agarose gel electrophoresis, in the presence or absence of hypoxia (250 μM CoCl2 for 24 h or 1% oxygen). Total RNA was extracted using RNA isolater Total RNA Extraction Reagent (Cat. No. R401-01; Vazyme Biotech Co., Ltd., Nanjing, China), and the extracted RNA reverse transcribed using HiScript II RT SuperMix (Cat. No. R223-01; Vazyme Biotech Co., Ltd., Nanjing, China). The 5′-gtatcaagtgctaccagagtgtc-3′ (forward) and 5′-cttatactgcatgacaagttcgc-3′ (reverse) primers were designed for PCR amplification of DGKG-Δ exon13. The amplified products of the U87-MG and T98G glioma cell lines before and after oxygen deprivation were subjected to agarose-gel electrophoresis and Sanger sequencing by Genewiz Biotech Co. Ltd. (Suzhou, China).

### Western blotting (WB)

WB was performed using rabbit anti-DGKG (1:1000, Cat. No. ab89037; Abcam, Cambridge, UK) and anti-β-actin (1:5000, Cat. No. ab8226; Abcam, Cambridge, UK) antibodies. The samples were lysed with RIPA lysis buffer (catalog number: P0013B; Beyotime Biotechnology Co., Ltd., Shanghai, China) containing protease inhibitors (SKU 11836153001, Roche, Basel, Switzerland). The lysates were loaded and separated by sodium dodecyl sulfate polyacrylamide gel electrophoresis (SDS-PAGE) using 10% gels (Cat. No. E303-01; Vazyme Biotech Co., Ltd., Nanjing, China). The separated protein bands were transferred to a polyvinylidene fluoride (PVDF) membranes (Cat. No. GVWP02500; MilliporeSigma, MA, USA) that were blocked by incubating with blocking buffer (P0233, Beyotime, Shanghai, China) for 1 h. The PVDF membranes were then incubated overnight with anti-DGKG antibody (1:1000) at 4°C. The membranes was then incubated with horseradish peroxidase (HRP)-conjugated secondary antibodies (1:10000; Cat. No. 7074; CST, USA) 27°C for 1 h, and the bands were spotted using an enhanced chemiluminescence (ECL) kit (Cat. No. PI32209; ThermoFisher Scientific, Waltham, MA, USA).

### Quantitative realtime-PCR (qRT-PCR)

Total RNA extraction and reverse transcription were performed as previously described for PCR. qRT-PCR was calculated using 2-ΔΔCT method. The following primers used for qRT-PCR: GAPDH-F: 5′-GTCTCCTCTGACTTCAACAGCG-3′;GAPDH-R: 5′-ACCACCCTGTTGCTGTAGCCAA-3′: DGKG-F: 5′-CGCTTCCATTGCACACAGATTC-3′ and DGKG-R: 5′-CTGCAAAAGTCTCCGAGGTGC-3′; MORC1-F: 5′-GAGAAGCAACTTAGAGAGTCGGTC-3′ and MORC1-R: 5′-GTTAGGCAGGTGGAAGTGACAG-3′; FGD3-F: 5′-GCTCAAGGACTATCTGAAGAGGC-3′ and FGD3-R: 5′-GAATGGCAGCATTGGAGTGGTTG-3′; KLHDC7B-F: 5′-GCACAACTACCTGTTTCTGGCG-3′ and KLHDC7B-R: 5′-TGGCTCCAGATGTTGGTCAGAG-3′; ATP1A2-F: 5′-CTGAACTTTCCCACGGAGAAGC-3′ and ATP1A2-R: 5′-GCCTTGGCTGTGATAGGGTG-3′; INHBE-F: 5′-CCCAGAATAACTCATCCTCCACC-3′ and INHBE-R: 5′-GGACAGGTGAAAAGTGAGCAGG-3′; TMEM119-F: 5′-GGATAGTGGACTTCTTCCGCCA-3′ and TMEM119-R: 5′-GGAAGGACGATGGGTAATAGGC-3′; CCN2-F: 5′-CTGCAGGCTAGAGAAGCAGAG-3′ and CCN2-R: 5′-GCTCAAACTTGATAGGCTTGGAG-3′; EFNB3-F: 5′-AGTTCCGCTCGCACCACGATTA-3′ and EFNB3-R: 5′-CACTCGGAGAAGCACCTTCATG-3′; IL16-F: 5′-TTGGACACAGGGTTCTCGCTCA-3′ and IL16-R: 5′-AGCAGGGAGATAACGGACTGAC-3′.

### Construction of lentiviral vector and infection

Lentiviral vectors for DGKG knockdown (KD), and vectors overexpressing (OE) DGKG or DGKG-Δ exon13 were constructed by Hanyin Biotech Ltd., Co. (Shanghai, China). The sequences used for DGKG knockdown were: KD1: 5′-GTGGGAGCCTCAAACAATA-3′; KD2: 5′-CACCGCAAATGTGAATTAT-3′ and KD3: 5′-GGGTGGACCTGAGCAACAT-3′.

### Cell proliferation assays

U87-MG and T98G cells, with or without DGKG knockdown or over expression, and cells expressing DGKG-Δ exon13, were seeded in 96-well plates at a density of 3000 per well. Then 10 μL of CCK-8 reagent (Cat. No. CK04-01; Dojindo Laboratories, Kumamoto, Japan) was added to the cells. The absorbance was measured at 450 nm (OD450) on days 1, 2, 3, 4, and 5 using a 96-well plate reader (ELX808, BioTek, USA). The experiments were performed at least in triplicate.

### Transwell and matrigel-transwell experiments

U87-MG and T98G cells, with or without DGKG knockdown or overexpression, and cells expressing DGKG-Δ exon13 were seeded in the upper chamber of a Transwell insert at a density of 1500–2000 cells per well. MEM or DMEM medium supplemented with 10% FBS was added to the bottom chamber. The top layer was cleaned with a sterilized cotton swab to remove the residual cells following overnight incubation. The cells that had migrated were stained with 0.1% crystal violet for 30 min and enumerated. The cell count was determined from five random fields per well, and the average number of cells was calculated. The assays were conducted in triplicate. The difference between Transwell and Matrigel-Transwell assays is that the upper chamber is coated with Matrigel in Matrigel-Transwell assays (catalog number CLS3422; 8-µm pores, MilliporeSigma).

### Construction of orthotropic tumor models

The animal studies were approved by the Institutional Animal Care and Use Committee of the Guizhou Medical University. A total of 24 athymic male nu/nu mice, aged 6-weeks were obtained from Lingchang Biotech, Shanghai, China and used to construct the murine model of GBM. Then 5 × 10^5^/5 μL of the control U87-MG cells and the constructed U87-MG-DGKG-KD3, U87-MG-DGKG-KD3/DGKG-wildtype (WT)-recue (RES), and U87-MG DGKG-KD3/DGKG-Δ exon13-RES cells were implanted in the *corpus* striatum of anaesthetized athymic nude mice using a stereotactic frame (David Kopf Instruments). The mice were monitored daily and subjected to examination by magnetic resonance imaging MRI scanning when weight loss or neurologic impairments were observed. The diameters of the tumors were measured from the MRI scans and the tumor volumes were calculated using the formula: (length × width^2^)/2, with the Function Analysis software (General Electric Company, USA). The mice were operated on and housed according to the criteria outlined in the Guide for the Care and Use of Laboratory Animals of Guizhou Medical University. The mice were randomly assigned to different groups with 8 mice per group.

### Transcriptome sequencing

A total of 2 × 10^7^ U87MG-negative control (NC) (infected with control lentivirus), U87-DGKG-KD, U87-DGKG-OE, and U87-DGKG-Δ exon13-OE cells were collected, and the total RNA was extracted using TRIzol reagent. The poly (A) mRNA was isolated using Oligo (dT) beads. The samples were loaded on an Illumina Novaseq or MGI2000 platform for sequencing using a 2 × 150 paired-end configuration according to manufacturer’s instructions (Genewiz, China).

### Statistical analyses

The data obtained from at least three independent experiments were subjected to statistical analyses using two-tailed Student’s *t*-test, and two-way analysis of variance (ANOVA). The data obtained from the experiments were analyzed with GraphPad Prism software, version 9 (Graphpad, USA). Two-sided *p* values less than 0.05 were considered to be statistically significant.

## Results

### Hypoxia-induced expression of the alternatively spliced DGKG-Δ exon13 transcript in several GBM cells

**Figure 1:**
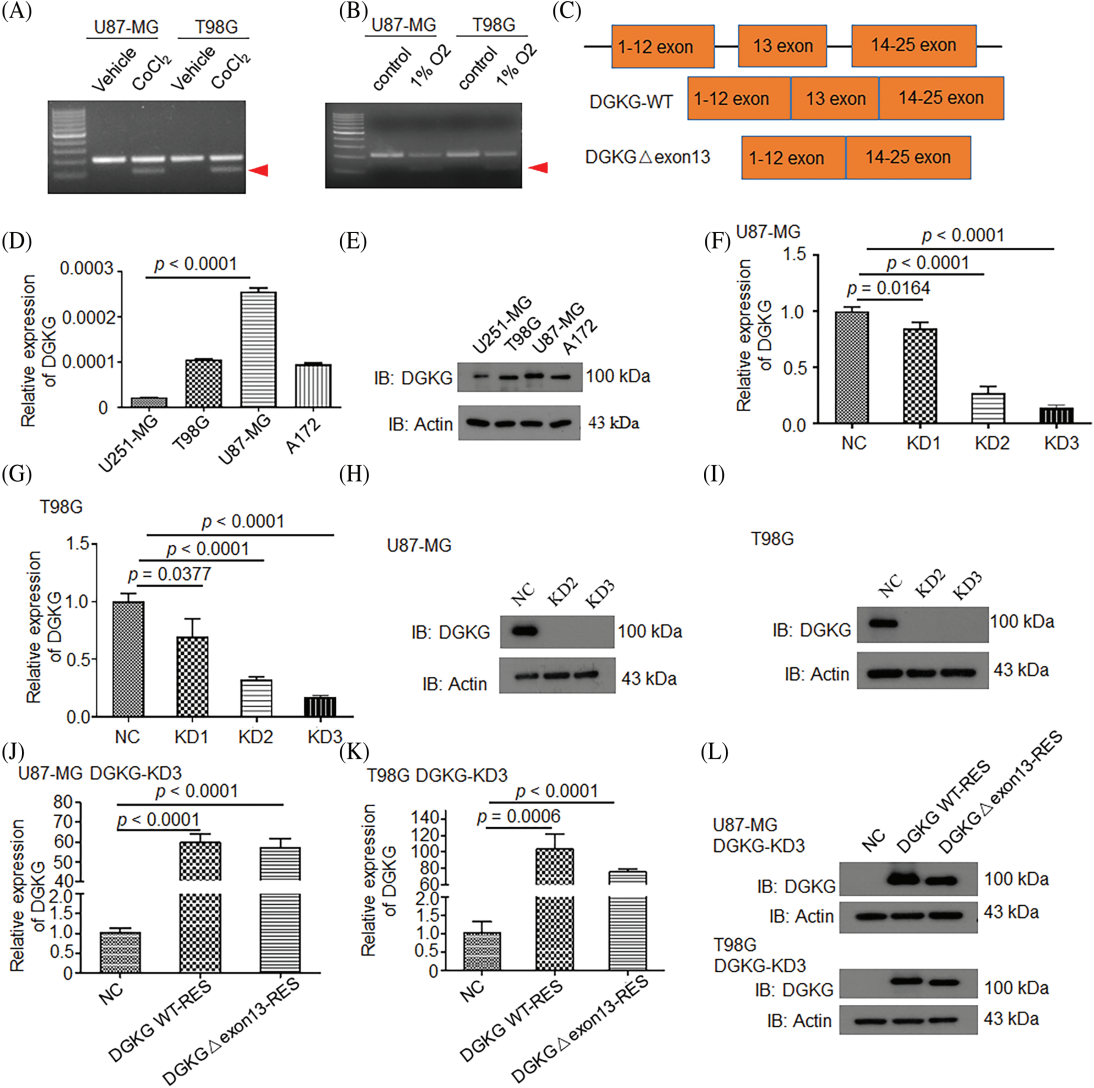
Hypoxia induced the generation of the alternatively spliced DGKG-Δ exon13 transcript in GBM cells. (A, B). Analysis of the expression of the wild-type DGKG and alternatively spliced DGKG-Δ exon13 in U87-MG and T98G cells by agarose gel electrophoresis, in the presence or absence of hypoxia (250 μM CoCl_2_ or 1% O_2_). (C). Verification of the sequences of wild-type DGKG and DGKG-Δ exon13 transcripts by Sanger sequencing. (D). Analysis of the mRNA levels of DGKG in glioma cells by RT-PCR. (E). Analysis of the levels of DGKG protein in glioma cells by WB. (F–I). Knockdown of DGKG expression in U87-MG (F, H) and T98G (G, I) cells. (J–L). Expression of wild-type DGKG and DGKG-Δ exon13 in U87-MG-DGKG-KD3 (J, L) and T98G-DGKG-KD3 (K, L) cells.

The results of agarose gel electrophoresis revealed that the DGKG gene produced alternatively spliced variants in U87-MG and T98G cells under hypoxia simulated by CoCl_2_ treatment (Fig. 1A) or 1% oxygen (Fig. 1B), and Sanger sequencing revealed exon 13 of DGKG underwent alternative splicing (Fig. 1C). The expression levels of DGKG mRNA and protein were detected in several glioma cells and the results demonstrated that DGKG was expressed in glioma cells, especially in radiotherapy-resistant T98G cells (Fig. 1D,E). T98G and U87-MG cell lines were selected for establishing the DGKG-knockdown cells, and the efficiency of knockdown was validated at both the protein and mRNA levels (Fig. 1F–I). DGKG WT-RES and DGKG-Δ exon13-RES U87-MG and T98G cells were additionally established using the DGKG knockdown cells and expression levels of DGKG mRNA and protein were subsequently validated (Fig. 1J–L).

The findings therefore revealed that hypoxia induced the generation of the alternatively spliced DGKG-Δ exon13 transcript, whose functions remain unknown.

### DGKG-Δ exon13 promoted the proliferation, migration, and invasion of U87-MG and T98G cells

**Figure 2:**
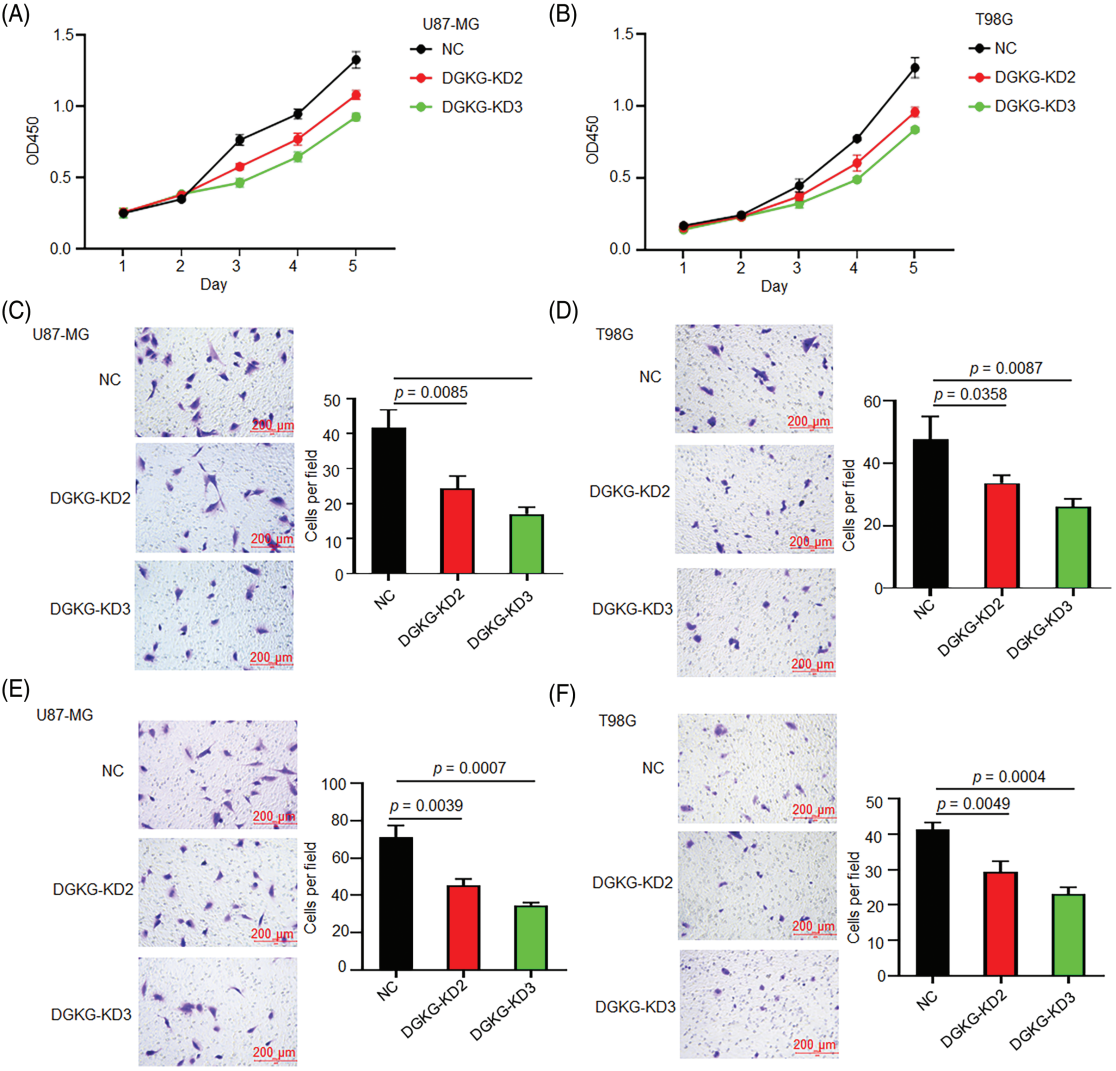
DGKG knockdown reduced the proliferation, migration, and invasion of U87-MG and T98G cells. (A, B). The proliferative ability of U87-MG and T98G cells decreased significantly following DGKG knockdown, as revealed (3 times) by CCK-8 assays. (C, D). DGKG knockdown in U87-MG and T98G cells significantly weakened their migratory potential, as revealed (3 times) by Transwell assays. (E, F). DGKG knockdown in U87-MG and T98G cells significantly reduced their invasive potential, as revealed (3 times) by Matrigel-Transwell assays. NC was cells infected with control lentivirus.

The results of CCK-8 assays revealed that the proliferative ability of U87-MG and T98G cells decreased significantly following DGKG knockdown (Fig. 2A,B). The results of Transwell and Matrigel-Transwell assays revealed that the knockdown of DGKG in U87-MG and T98G cells markedly weakened their migratory and invasive potential (Fig. 2C–F).

**Figure 3:**
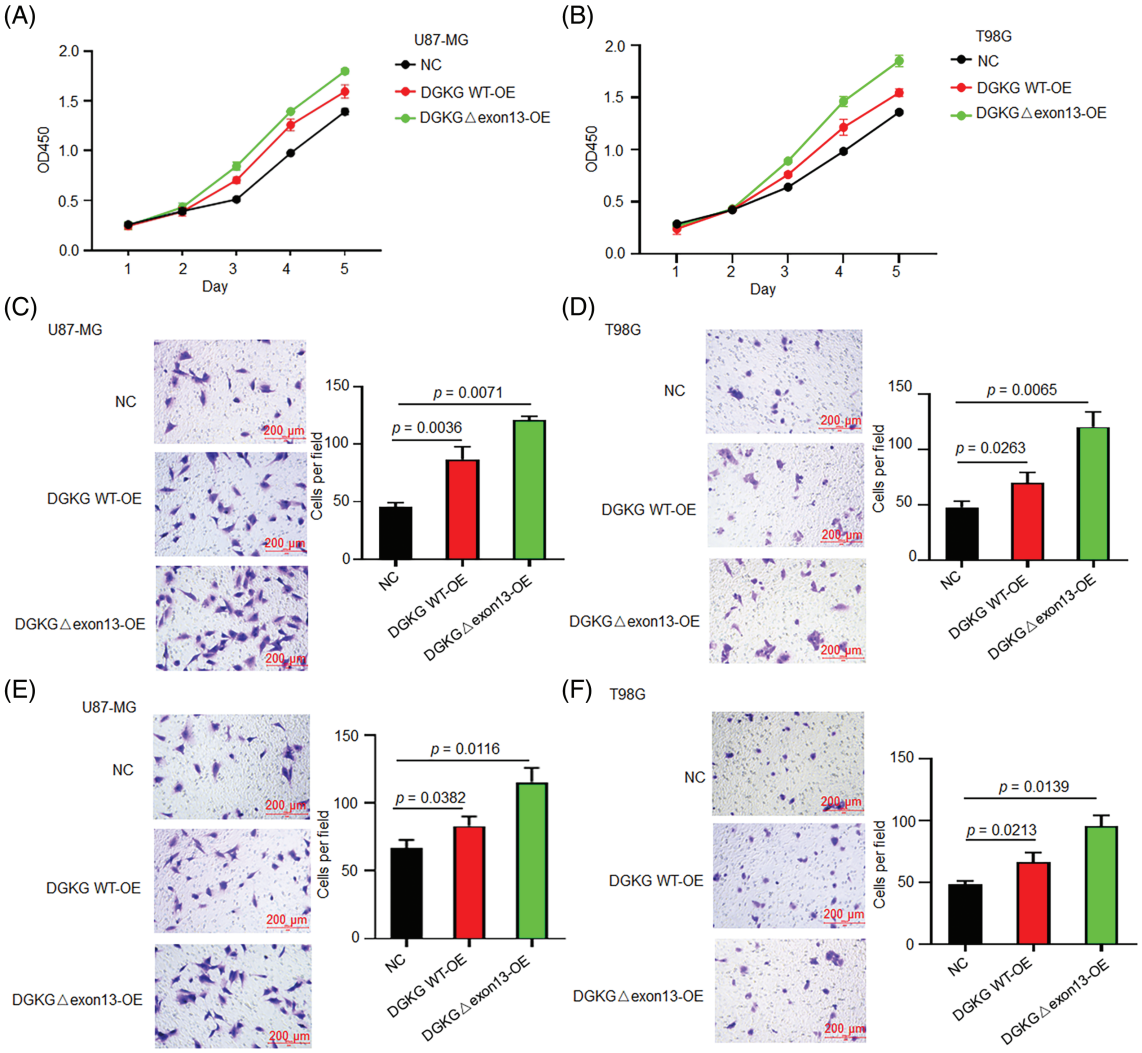
DGKG-Δ exon13 promoted the proliferation, migration, and invasion of U87-MG and T98G cells. (A, B). The proliferative ability of U87-MG and T98G cells with DGKG-Δ exon13 RES was greater than that of cells expressing the wild-type DGKG gene, as revealed by CCK-8 assays. (C, D). The migration potential of U87-MG and T98G cells with DGKG-Δ exon13 RES was significantly higher than that of cells expressing the wild-type DGKG gene, as revealed by Transwell assays. (E, F). Matrigel-Transwell assays revealed that the invasive potential of U87-MG and T98G cells with DGKG-Δ exon13 RES increased significantly compared to that of cells expressing the wild-type DGKG gene.

In contrast, the proliferative ability of U87-MG and T98G cells with DGKG-Δ exon13 RES was greater than that of the cells expressing the wild-type DGKG gene (Fig. 3A,B). The stable expression of the wild-type DGKG gene or DGKG-Δ exon13 in U87-MG and T98G cells promoted migration and invasion. The migration and invasion potential of U87-MG and T98G cells with DGKG-Δ exon13 RES significantly compared to that of cells expressing the wild-type DGKG gene (Fig. 3C–F).

Altogether, the results of functional studies revealed that DGKG, and especially DGKG-Δ exon13, promoted the proliferation, migration, and invasion of glioma cells *in vitro*.

### DGKG-Δ exon13 promoted the progression of GBM in vivo

**Figure 4:**
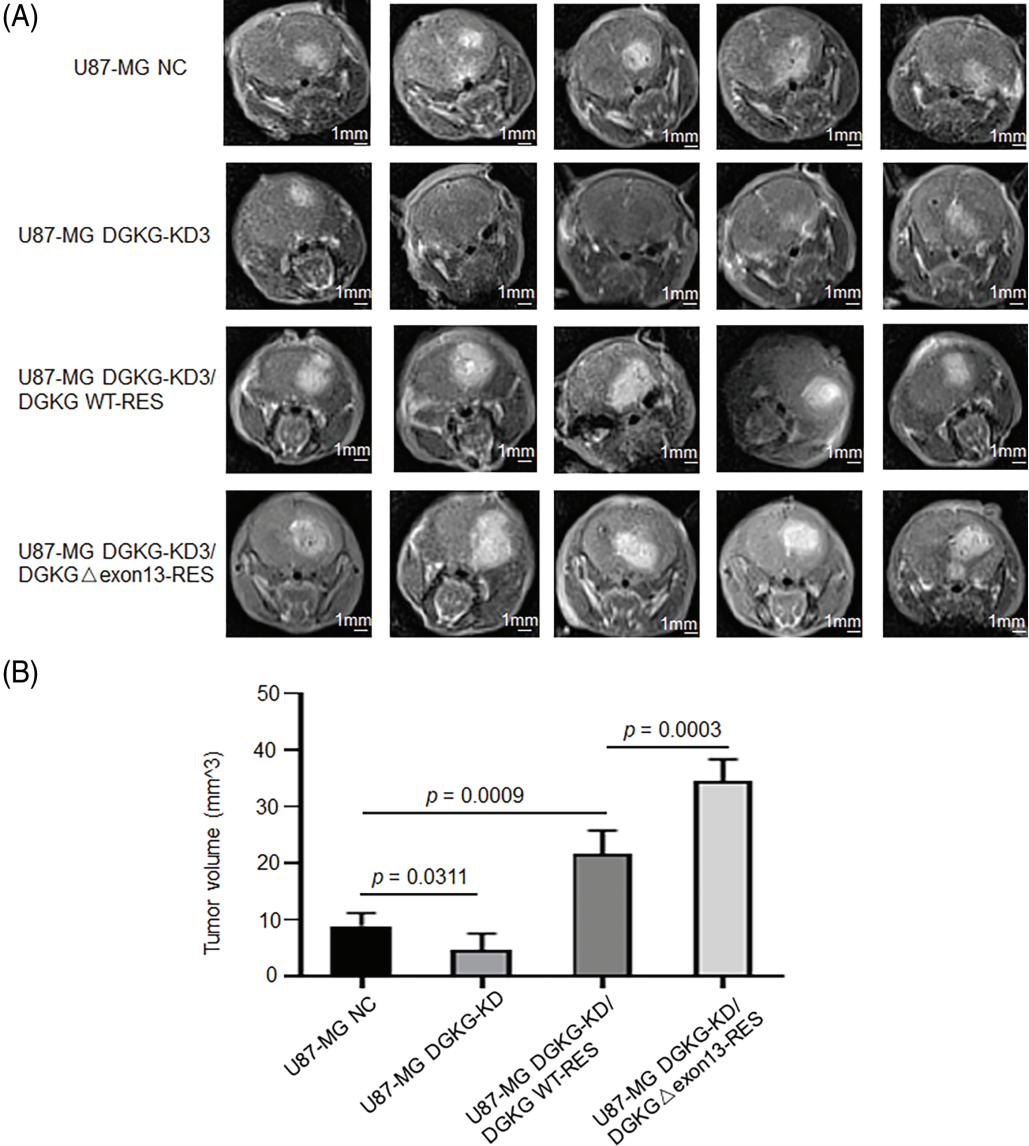
DGKG-Δ exon13 promoted the progression of GBM *in vivo*. (A). Representative MRI scans of mice implanted with the constructed U87-MG cell lines (negative control lentivirus (NC), DGKG-KD3, DGKG-KD3/DGKG WT-RES, and DGKG-KD3/DGKG-Δ exon13 RES). (B). Tumor volumes in the aforementioned groups.

The *in vivo* functions of DGKG and DGKG-Δ exon13 were further confirmed using orthotopic glioma cancer models, established from the U87-MG cell lines (NC, DGKG-KD3, DGKG-KD3/DGKG WT-RES, and DGKG-KD3/DGKG-Δ exon13 RES) constructed herein (Fig. 4A).

The results of MRI scanning revealed that the tumor volumes in the DGKG-KD3 group were much smaller than those of the negative control (NC); however, the tumor volumes were restored in the DGKG and DGKG-Δ exon13 rescue groups and were especially prominent in the DGKG-Δ exon13 rescue group (Fig. 4B).

Altogether, the functional studies revealed that DGKG, and especially DGKG-Δ exon13, promoted the progression of GBM *in vivo*.

### Identification of genes altered by DGKG knockdown or those specifically regulated by DGKG-Δ exon13

**Figure 5:**
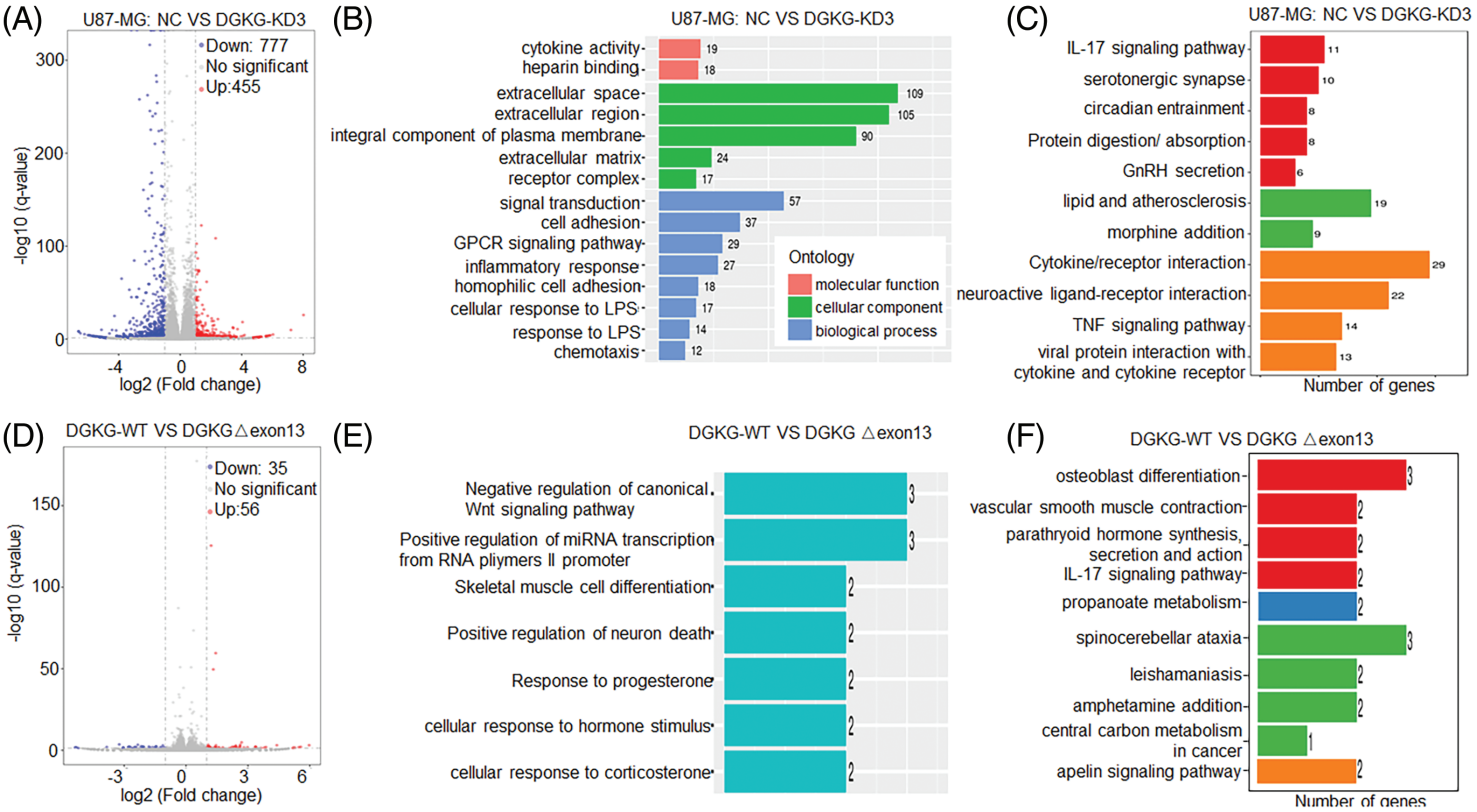
Identification of genes differentially expressed after DGKG knockdown or those specifically regulated by DGKG-Δ exon13, through transcriptome analyses. (A). Volcano plot depicting the genes that were differentially expressed in the U87-DGKG-KD group, of which 777 and 455 genes were downregulated and upregulated, respectively. (B, C). Results of GO and KEGG enrichment analysis of the differentially expressed genes in the U87-DGKG-KD group. (D). Volcano plot depicting the genes that were specifically regulated by DGKG-Δ exon13, of which 35 and 56 genes were downregulated and upregulated, respectively. (E, F). Results of GO and KEGG enrichment analyses of the differentially expressed genes regulated by DGKG-Δ exon13.

In order to investigate the mechanism by which DGKG affects the progression of GBM, the U87MG-NC and U87-DGKG-KD cells were subjected to transcriptome sequencing. The results demonstrated that compared to those of the U87-NC group, a total of 1232 genes were differentially expressed in the U87-DGKG-KD group, of which 777 genes and 455 genes were downregulated and upregulated, respectively (Fig. 5A). Additionally, the results of GO and KEGG enrichment analyses revealed that these differentially expressed genes were enriched in the extracellular space, signal transduction, IL-17 signaling pathway, lipid and atherosclerosis, and cytokine/receptor interaction terms, among others (Fig. 5B,C, Fig. S1).

The differentially expressed genes between DGKG and DGKG-Δ exon13 groups were additionally investigated. The findings revealed that compared to those of the WT-DGKG group, a total of 91 genes were altered in DGKG-Δ exon13, of which 35 and 56 genes were downregulated and upregulated, respectively (Fig. 5D). GO and KEGG enrichment analyses revealed that these differentially expressed genes were enriched in the negative regulation of canonical Wnt signaling pathway, osteoblast differentiation, vascular smooth muscle contraction, parathyroid hormone synthesis, secretion and action, and the IL-17 signaling pathway terms, among others (Fig. 5E,F, Fig. S2).

**Figure 6:**
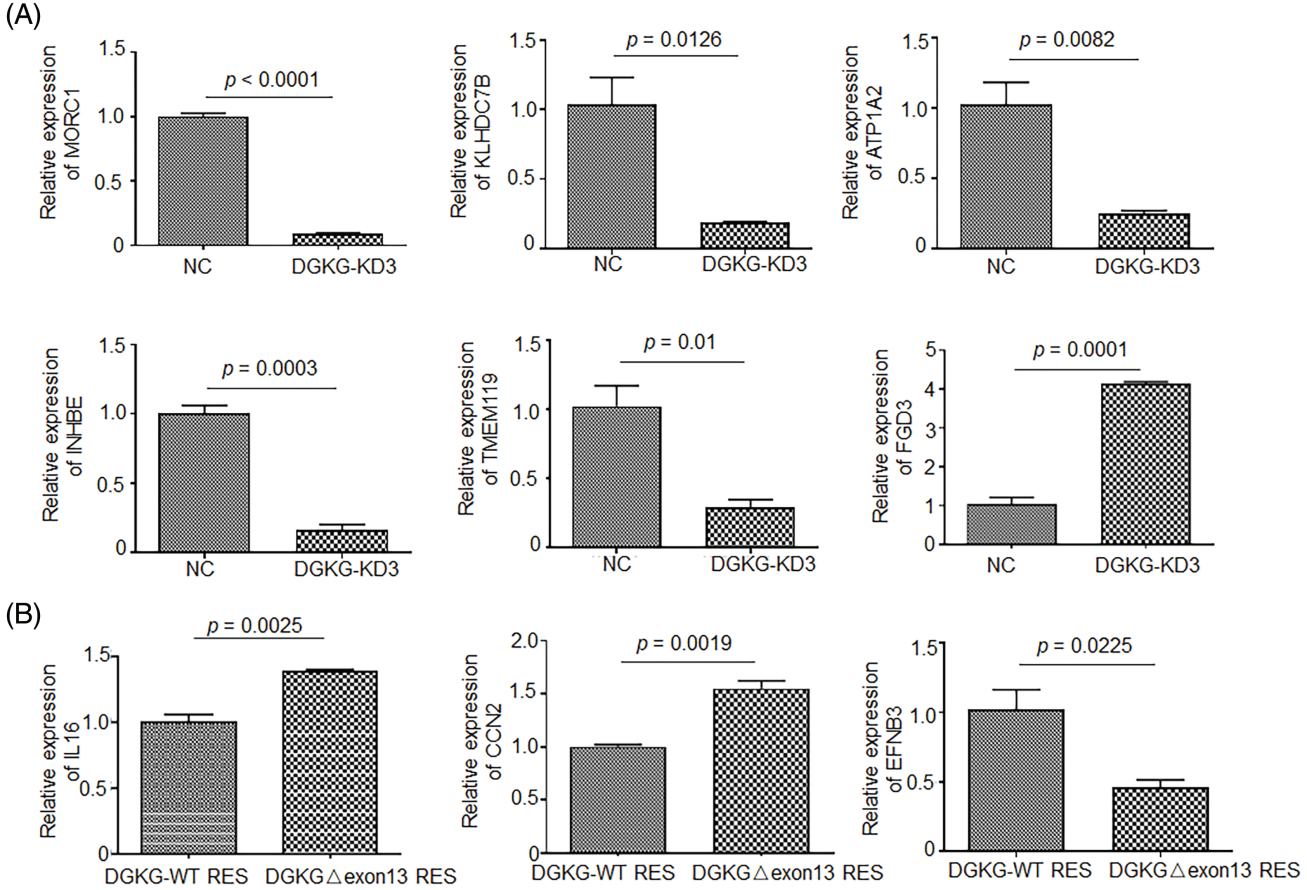
Relative expression levels of genes altered by DGKG knockdown or those specifically regulated by DGKG-Δ exon13. (A). Determination of the relative expression levels of MORC1, FGD3, KLHDC7B, ATP1A2, INHBE, and TMEM119 by qRT-PCR following DGKG knockdown in U87-MG cells. (B). Relative expression levels of CCN2, EFNB3, and IL16 in U87-DGKG-Δ exon13 and U87-DGKG-OE cells, as determined by qRT-PCR.

Based on the results of transcriptome sequencing, a total of 10 genes that underwent the most significant alterations, according to the fold change and *p* value, were selected. The MORC1, KLHDC7B, ATP1A2, INHBE, TMEM119, and FGD3 genes, which were significantly differentially expressed between the U87-DGKG-KD and NC groups, were selected herein. The results of qRT-PCR validation of MORC1, KLHDC7B, ATP1A2, INHBE, TMEM119, and FGD3 were consistent with the results of transcriptome sequencing (Fig. 6A).

The IL16, CCN2, and EFNB3 genes, which were differentially expressed between the U87-DGKG-Δ exon13 and U87-DGKG groups, were selected herein. The results of qRT-PCR validation of these genes were consistent with the results of transcriptome sequencing (Fig. 6B).

The results of transcriptome analysis, therefore, revealed that MORC1, KLHDC7B, ATP1A2, INHBE, TMEM119, and FGD3 altered significantly following DGKG knocked down and that IL-16, CCN2, and EFNB3 were specifically regulated by DGKG-Δ exon13.

## Discussion

The present study demonstrated that the DGKG-Δ exon13 transcript was generated in GBM tumor cell lines during hypoxia. The knockdown of DGKG decreased the proliferation, migration, and invasion potential of U87-MG and T98G cells, while DGKG-Δ exon13 significantly enhanced the progression of GBM *in vitro* and *in vivo*. The genes that were regulated by DGKG, and specifically by DGKG-Δ exon13 were identified by transcriptome analysis. The findings provide key insight into a likely potential novel target for the clinical treatment and diagnosis of GBM.

It has been reported that hypoxia promotes the phosphorylation of the Serine/arginine-rich splicing factor 1 (SF2/ASF) splicing factor via clk1, which in turn enhances the binding of SF2/ASF to the exon splicing silencer (ESS) cis-element near EES to induce the selective splicing of the oncogenic receptor tyrosine kinase RON. This leads to the skipping of exon 11 and the formation of the alternatively spliced RONΔ165 transcript. The constitutively active RONΔ165 transcript promotes the nuclear translocation of RON and β-catenin to induce the expression of downstream oncogenes, including c-Jun, c-Myc, and Cyclin D, which promote cellular proliferation [29]. The angiogenic factor vascular endothelial growth factor A (VEGF-A) undergoes alternative splicing under hypoxic conditions to produce the partially soluble VEGF-A165 variant. Due to its high affinity for heparin, VEGF-A165 promotes the co-expression of neuropilin and VEGFR to enhance the binding of VEGF to VEGF receptor (VEGFR), thus enhancing its efficiency as an angiogenic factor [30].

The present study is the first to report that DGKG and the novel DGKG-Δ exon13 promote the development of GBM. The findings indicated that DGKG could be a potential therapeutic target for GBM. However, the limitations of our study such as the molecular mechanism by which DGKG promotes the development of GBM were only preliminarily explored herein, and further studies are necessary to obtain deeper insights into its application in the treatment and diagnosis of GBM. The discovery of abnormal alternative splicing in RNA processing has not only provided new insights into tumor pathogenesis but also provides novel therapeutic opportunities in targeting these aberrations in various ways (e.g., small molecules, splice-switching oligonucleotides (SSOs), and protein therapies) to modulate alternative RNA splicing or other RNA processing and modification mechanisms. It might be useful to correlate the proteins involved in the treated pathways with the IDH and MGMT genes, trying to assign a prognostic value. Moreover, the mechanisms and functions of differential genes regulated by DGKG such as MORC1 [31], FDG3 [32], IL-16 [33], CCN2 [34] and EFNB3 [35] need further investigation.

## Conclusion

In summary, the present study revealed that DGKG undergoes alternative splicing under hypoxic conditions to generate a novel transcript lacking exon 13. DGKG promotes the development of GBM; however, the novel DGKG-Δ exon13 transcript, which is induced by a hypoxic microenvironment, has a more significant effect on promoting the growth and infiltration of GBM. The findings indicated that DGKG could serve as a potential therapeutic target for the intervention of GBM.

## Supplementary Materials

Figure S1 Results of GO enrichment analysis of the differentially expressed genes in the U87-DGKG-KD group.

Figure S2 Results of KEGG enrichment analysis of the differentially expressed genes regulated by DGKG-△exon13.

## Data Availability

All data are available from the corresponding author upon reasonable request.

## Supplementary Materials

The supplementary material is available online at https://doi.org/10.32604/or.2024.055102.
