# Supplementary figures and images for: The alternatively spliced diacylglycerol kinase gamma-Δ exon13 transcript generated under hypoxia promotes glioblastoma progression
Source: Oncol Res. 2025 Apr 18;33(5):1189–98. doi: 10.32604/or.2024.055102 (PMC12034013; doi:10.32604/or.2024.055102)

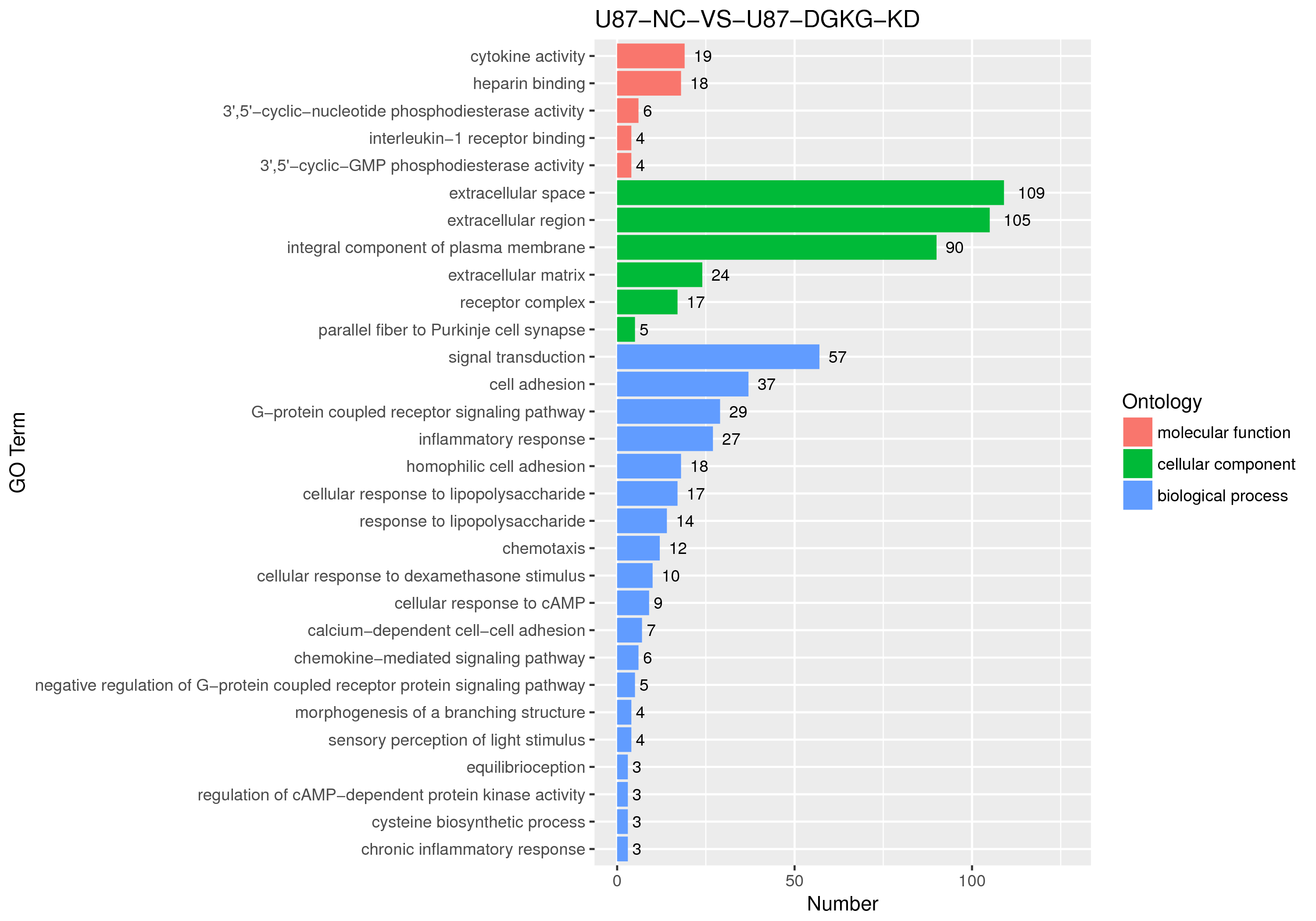

Supplement: Figure S1 [file OncolRes-33-55102-s001.png]

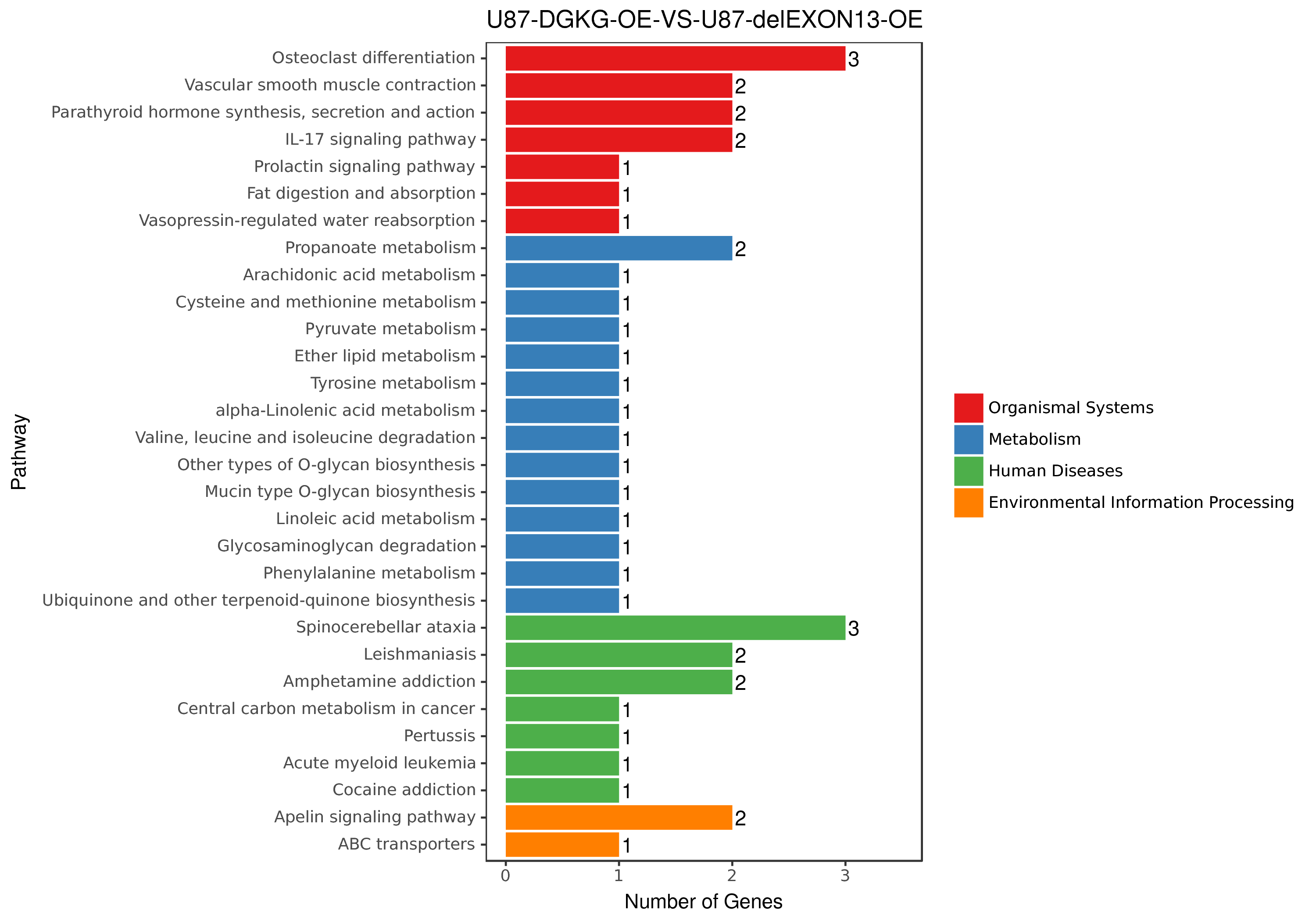

Supplement: Figure S2 [file OncolRes-33-55102-s002.png]
